# Mixed dentition space analysis among Nepalese Brahmins/Chhetris

**DOI:** 10.1186/s12903-016-0265-1

**Published:** 2016-08-02

**Authors:** Rajesh Gyawali, Basanta Kumar Shrestha, Rajiv Yadav

**Affiliations:** 1 Department of Orthodontics, B.P. Koirala Institute of Health Sciences, Dharan, Nepal; 2 Department of Dentistry, Institute of Medicine, Kathmandu, Nepal

**Keywords:** Mixed dentition, Nepalese Brahmins/Chhetris, Moyers method, Tanaka and Johnston method

## Abstract

**Background:**

Mixed dentition space analysis forms an important part of orthodontic diagnosis and treatment planning. Regression equations are widely used for mixed dentition analysis which can vary among races. This study aimed to find out the new regression equation in estimating the size of unerupted canines and premolars for Nepalese Brahmins/Chhetris.

**Methods:**

Hundred Nepalese Brahmins/Chhetris (50 males and 50 females) who met our criteria were selected among the patients attending to the Orthodontic Out-Patient Department, Institute of Medicine, Kathmandu. The mesiodistal widths of all mandibular permanent incisors; maxillary and mandibular canines and premolars were measured and analyzed. The results were also compared with predicted values from the Moyers and the Tanaka and Johnston methods. Correlation and linear regression analyses were performed between the predicted and actual tooth sizes for Nepalese Brahmins/Chhetris and standard regression equations were developed.

**Results:**

No significant differences were observed when the sum of canine and premolars of one quadrant is compared between sides and sex. Significant and high positive correlations were found between the mandibular incisors and the combined mesiodistal widths of the canines and premolars for the maxillary (*r* = 0.72) and mandibular (*r* = 0.73) segments. Significant differences were observed between the measured values from this study and from Moyers (50 % and 75 % probability) and Tanaka-Johnston methods.

**Conclusions:**

The equations and charts commonly used for North American children (50^th^ or 75^th^ percentile) did not accurately predict for our sample, so new regression equations and tables were developed for Nepalese Brahmins/Chhetris children.

## Background

Transition from deciduous to permanent dentition begins once permanent incisors and the first permanent molars erupt, which is known as the mixed dentition phase. Treatment planning in mixed dentition is more challenging because it requires prediction of future space requirement. Mixed dentition analysis forms an important aspect of orthodontic diagnosis and treatment planning. It is a valuable tool in determining whether the treatment plan involves serial extraction, guidance of eruption, space maintenance, space regaining or just the periodic surveillance [1, 2].

Mixed dentition analysis is concerned with the estimation of mesiodistal crown width of the permanent canines and premolars which can be done by measuring teeth size on the radiographs [3, 4], using regression equation, or measurement of erupted teeth as well as radiographs of unerupted teeth [5–9]. Accurate prediction can be done by measurements of mesiodistal width of these teeth on radiographs as well as on the cast [10, 11]. However, it requires obtaining both dental casts and radiographs for the analysis.

The commonly used Moyers mixed dentition analysis [12], and Tanaka-Johnston analysis [13] were developed from North European and North American Caucasian population which may not be applicable to other races [14–25]. A study by Jaiswal et al. [26] in the Nepalese population also showed that Moyers and Tanaka-Johnston method also didn’t accurately predict for Nepalese population. This study considered only male and female with no distinction among the various ethnic origin of the samples. As Nepal is a country with population of diverse ethnic origins [27], consideration of ethnicity is important in a mixed dentition space analysis. So, the purpose of conducting this study is to develop a prediction method for Nepalese Brahmins/Chhetris comprising nearly one third of the total population.

## Methods

It is a cross sectional, analytical study done at Orthodontics and Dentofacial Orthopedic Unit, Department of Dentistry, Institute of Medicine, Tribhuvan University Teaching Hospital, Kathmandu, Nepal after the ethical clearance from its own Institutional Review Board. All the study models made as a part of diagnostic records for patients seeking orthodontic treatment were utilized in this study.

Inclusion criteria were:

- Nepalese Brahmins/Chhetris with Brahmins/Chhetris ancestors at least from one previous generation identified through the history,
- All permanent teeth till first molar erupted.

Exclusion criteria were:

- Interproximal caries or restorations,
- Missing or supernumerary teeth,
- Abnormally sized or shaped teeth determined clinically,
- Significant teeth wear seen clinically,
- Previous orthodontic treatment.

During calculation of sample size, following points were considered.

- Confidence level of 95 %
- Significance level of 0.05
- Statistical power of 80 % [28]
- Clinically important smallest difference of 0.2 mm [29, 30]
- Standard deviation of the measurement 0.3 mm [20, 31]

The sample size was calculated as-

$$ Standarized\; difference=\frac{Smallest\; relevant\; difference}{Estimated\; standard\; deviation} $$

$$ =\frac{0.2}{0.3}=0.6 $$

So, joining the standardized difference with power of 0.8 at the significance level of 0.05 in Altman Nomogram, the sample size was ≈ 90. Hence, a sample size of 100 was taken (50 males and 50 females).

The mesiodistal width of the individual teeth was measured by digital caliper-Shan (Guilin Measuring and Cutting Tools Co. Ltd, Guangxi, China). Teeth were measured to the nearest 0.01 mm. To get better access to the proximal areas, fine pointed wire segments were attached to the tip of the caliper. The mesiodistal dimensions of the teeth were determined by measuring the maximum distance between approximate surfaces of the teeth as described by Hunter and Priest [32].

Single investigator (RG) measured all the casts with the digital caliper. To determine the intra-observer reliability, 20 study models were randomly selected (systematic random sampling) and remeasured after 2 weeks. Intraclass Correlation Coefficient (ICC) with absolute agreement was calculated to be 0.94 indicating high reliability.

For each patient, the sum of following groups of teeth was calculated -

- Four mandibular incisors
- Mandibular canine and premolars in each quadrant
- Maxillary canine and premolars in each quadrant

The sum of the four permanent mandibular incisors was used to predict the combined size of the permanent unerupted canines and premolars using both Moyers method and Tanaka –Johnston method.

## Results

The values of Kolmogorov-Smirnov and Shapiro Wilk tests were above 0.05 proving the normal distribution of the data (mesiodistal dimension of mandibular incisors, canines and premolars). The sample had a mean age of 18.28 and 17.98 years for male and female respectively. When the combined mesiodistal width of permanent canines, first and second premolars of either side of the dental arch was compared, there was no statistically significant difference between the right and the left side. T-test was done to detect the gender differences in sum of mesiodistal width of canine and premolars. It was found that there is no statistically significant gender difference when comparison was done between the mean sum of canine and premolars.

T-test was employed to compare the difference between the measured and predicted values derived from Moyers and Tanaka-Johnston methods for maxillary and mandibular arches for both male and female. Statistically significant differences were observed between the measured values from this study and from Moyers method (except for female mandibular arch at 50 % probability and male maxillary arch at 75 % probability). Tanaka Johnston method significantly over-estimated the values in both maxillary and mandibular arch (Table 1).

**Table 1:** Comparison of actual and predicted values

Arch	Prediction method	Mean difference (Actual –predicted)	SD	SEM	95 % Confidence Interval of the Difference			
					Lower	Upper	t	Sig (2-tailed)
Maxilla (female)	Moyers 75 %	0.40	1.14	0.16	0.08	0.73	2.51	0.016*
	Moyers 50 %	1.15	1.13	0.16	0.83	1.47	7.20	0.000*
Mandible (female)	Moyers 75 %	-0.31	0.94	0.13	-0.57	-0.04	-2.30	0.026*
	Moyers 50 %	0.22	0.95	0.13	-0.05	0.48	1.61	0.115
Maxilla (male)	Moyers 75 %	-0.11	0.90	0.13	-0.37	0.15	-0.86	0.395
	Moyers 50 %	0.44	0.90	0.13	0.18	0.69	3.45	0.001*
Mandible (male)	Moyers 75 %	-0.40	0.97	0.14	-0.67	-0.12	-2.89	0.006*
	Moyers 50 %	0.38	0.96	0.14	0.11	0.66	2.80	0.007*
Maxilla	Tanaka-Johnston	-0.84	0.97	0.14	-1.12	-0.57	-6.13	0.000*
Mandible		-0.97	0.95	0.13	-1.24	-0.69	-7.16	0.000*

*SD* Standard Deviation, *SEM* Standard Error of Mean

* : *p* < 0.05, significant

The regression characteristics of the obtained prediction equations are presented (Table 2). The accuracy of prediction is often expressed as the standard error of mean of the prediction equations. In this study, the standard error of estimates (mean) ranged between 0.88 to 0.93 mm for male, female and both sexes. The Pearson product moment correlation coefficient (r) is above 0.6 and can be put into clinical orthodontic use [33] by constructing regression equations for Nepalese Brahmins/Chhetris (Table 3).

**Table 2:** Regression characteristics

Canine-premolar segment	Sex	*r*	*r* ^*2*^	Regression coefficient		Standard error of mean (mm)	95 % Confidence interval
				a	B		
Maxillary	M	0.676	0.457	8.911	0.563	0.905	0.385 to 0.742
	F	0.767	0.588	3.650	0.777	0.905	0.588 to 0.966
	M + F	0.719	0.517	6.335	0.668	0.916	0.538 to 0.797
Mandibular	M	0.772	0.596	4.603	0.729	0.884	0.554 to 0.903
	F	0.705	0.498	5.583	0.668	0.934	0.474 to 0.863
	M + F	0.734	0.539	4.898	0.707	0.927	0.576 to 0.838

*r* Correlation coefficient, *r* ^*2*^ Coefficient of determination, *M* Male, *F* Female

**Table 3:** Regression equations of Nepalese Brahmins/Chhetris

Arch	Sex	Equation
Maxillary	M	y = 8.91 + 0.56x
	F	y = 3.65 + 0.78x
	M + F	y = 6.34 + 0.67x
Mandibular	M	y = 4.60 + 0.73x
	F	y = 5.58 + 0.67x
	M + F	y = 4.90 + 0.71x

*M* Male, *F* Female, *x* sum of mesiodistal dimension of four mandibular incisors, *y* Sum of mesiodistal dimension of canine and premolars

Using the prediction equation, prediction table is made for Nepalese Brahmins/Chhetris (Table 4).

**Table 4:** Prediction table for Nepalese Brahmins/Chhetris

LI (mm)	Male		Female	
	∑ CP1P2 Maxilla (mm)	∑ CP1P2 Mandible (mm)	∑ CP1P2 Maxilla (mm)	∑ CP1P2 Mandible (mm)
18.0	19.0	17.7	17.5	17.6
18.5	19.3	18.1	17.9	17.9
19.0	19.6	18.5	18.3	18.3
19.5	19.9	18.8	18.7	18.6
20.0	20.2	19.2	19.1	18.9
20.5	20.5	19.5	19.4	19.3
21.0	20.7	19.9	19.8	19.6
21.5	21.0	20.3	20.2	19.9
22.0	21.3	20.6	20.6	20.3
22.5	21.6	21.0	21.0	20.6
23.0	21.9	21.4	21.4	20.9
23.5	22.1	21.7	21.7	21.3
24.0	22.4	22.1	22.1	21.6
24.5	22.7	22.5	22.5	21.9
25.0	23.0	22.8	22.9	22.3
25.5	23.3	23.2	23.3	22.6
26.0	23.5	23.6	23.7	23.0
26.5	23.8	23.9	24.1	23.3
27.0	24.1	24.3	24.4	23.6
27.5	24.4	24.7	24.8	24.0
28.0	24.7	25.0	25.2	24.3

LI – Sum of mesiodistal dimension of lower incisors

∑ CP1P2 – Sum of mesiodistal dimension of canine and two premolars

## Discussion

Various mixed-dentition analyses are reported in the literature (regression equations, radiographic methods, or combination of both), but the regression equations based on measurements from already erupted permanent teeth in the early mixed dentition are the most widely used. Therefore, our study was conducted to corroborate their principles in a Nepalese Brahmins/Chhetris sample.

The study sample selection did not exclude patients with crowding as the study was conducted in patients coming to the Orthodontic Out Patient Department seeking orthodontic treatment. Patients with crowding have been shown to have no difference in mesiodistal tooth dimension compared to those with no crowding [34]. According to Puri et al. [35], mesiodistal crown dimension of individual teeth, sum of incisors and the sum of canines and premolars are uniformly larger in crowded arches as compared to normal and spaced dentition; but the correlation of the combined mesiodistal crown dimension of incisors with the combined mesiodistal crown dimension of canines and premolars was significantly positive in crowded, spaced and normal dentition.

The absolute mean difference between the left and right mesiodistal width of any individual tooth ranged from 0.01 to 0.07 mm. The mandibular central incisor showed least difference whereas maxillary canine the maximum. However, they are not statistically significant. Previous odontometric studies [36, 37] also have shown that there is no significant bilateral difference in mesiodistal tooth size. However, according to the study by Shrestha R [31] in Nepalese dentition, some teeth are significantly larger on the right side, which include- maxillary first molars, maxillary second molars, mandibular central incisors, and mandibular lateral incisors.

In this study, the mean mesiodistal tooth widths of male subjects was found to be larger than that of female in both maxillary and mandibular dental arches but only mandibular canine showed statistically significant difference. This result is in accordance with the study by Shrestha R [31] who found that all mesiodistal crown dimensions of Nepalese males are wider than those of the females but he found that maxillary canine, mandibular canine and mandibular lateral incisors had statistically significant difference.

Similarly, Seipel [38] found maximum sex difference for deciduous and permanent canines. Also, Staley and Hoag [5] found that the width of male canines was significantly larger than the canine width of the females. According to Doris et al. [39], North American Caucasian males have larger teeth than female, particularly, the maxillary central incisors and canines and the mandibular canines and both premolars.

Various studies in different population have found that the average mesiodistal tooth width of the male is larger than that of the female such as by Arya et al. [40] and Staley & Hoag [5] in Northwest Europeans; Richardson and Malhotra [29] in American Negros; Axelsson and Kirveskari [41] in Icelanders; Bishara et al. [37] in North Mexican children.

In this study, when the sum of incisors, canine & premolars is considered, there is no statistically significant difference between male and female which is in accordance to the study done by Bherwani and Fida [20] in Pakistani samples and in contrast to that of by Yuen et al. [42] in Southern Chinese; Jaroontham and Godfrey [21] in Thai population; Diagne et al. [43] in Senegalese population.

Most studies have found the sum of the four mandibular incisors to be one of the best predictors in the linear regression equations for determining the combined mesiodistal width of the unerupted permanent canines and premolars [3, 12, 44].

There are several clinical advantages of using four permanent mandibular incisors in prediction equations because they-[12]

- Erupt into the mouth early in the mixed dentition
- Are easily measured accurately
- Show less variation in shape and size

The present study also used the dimension four permanent mandibular incisors as the independent variable.

It may not be possible to attain very high precision in predictive methods based on tooth size measurements on dental casts, though reasonable reliable prediction can benefit a mixed dentition patient and the orthodontist by assisting in the development of a sound diagnosis [9]. Moyers claimed that from the mandibular incisors on cast alone, 95 % of the patients have combined mesiodistal widths of canine and premolars within one millimeter of the predicted value in his table, which should be considered clinically acceptable.

If there are systematic differences in the dependent variable (sum of permanent canine and the two premolars in a quadrant) associated with the levels of the sum of the four mandibular incisors (predictor), these differences are attributed to the predictor. The goal of the present study is to examine the ability of the predictor to reproduce the values of the mesiodistal width of permanent canines, first and second premolars in one quadrant. The mean difference (residuals) between the predicted and measured values can be used as the measure of error of the predictions. The squared value of residuals or the absolute mean difference provides a measure of how good the prediction is. When the predictions are close to the measured values, the squared errors or the absolute mean differences are small.

Permanent teeth may be either wrongly retained or extracted on the basis of an inaccurate tooth size prediction. Underestimation of the mesiodistal tooth width would result in a more conservative clinical approach, while overestimation tends to exaggerate space requirements and result in unnecessary extractions. Theoretically, the 50 % probability level is used as the estimate in all regression equations since any error would be distributed equally in either direction [12]. Clinically the value at the 75 % level is used as the estimate because more protection on the down side (crowding) is required than that of on the up side (spacing). Seventy-five percent level of probability means that 75 times out of 100 the unerupted canine and premolars will be at the predicted value or less. Nevertheless, the choice of percentile levels to be used may vary among clinicians depending on the application and experience of the clinician [12].

Tanaka-Johnston method tends to overestimate the combined mesiodistal width of the unerupted permanent canines and the two premolars which is in accordance with the study done by Jaiswal et al. [26] in Nepalese population.

Several other studies in different population also found that the Tanaka-Johnston method tends to overestimate the unerupted canine and premolars, such as the study by Al Bitar et al. [22] in Jordanian, Arslan et al. [45] in Turkish, Burhan and Nawaya [46] in Syrians, Buwembo et al. [47] in Ugandan population. However, Jaroontham & Godfrey [21] in Thai population and Ngesa [48] in Kenyan found Tanaka-Johnston equation close to the actual measurements.

In our study, Moyers prediction at 50 % probability tends to underestimate the combined mesiodistal width of the unerupted permanent canine and premolars in both male and female. But Jaiswal et al. [26] found that Moyers at 50 % probability tend to underestimate in males and overestimate in females.

Similarly, various authors also found that Moyers at 50 % tend to underestimate the size of unerupted canine and premolars, such as the study by Jaroontham and Godfrey [21] in Thai population, Melgaco et al. [49] in White Brazilians, Buwembo et al. [47] in Ugandan population, Paredes et al. [16] in Spanish sample.

The standard error of the estimate (SEE) is a measure of the accuracy of predictions. The SEE of this study are 0.92 and 0.93 for maxillary and mandibular arch respectively. These values are comparable to the errors presented in the literature (Table 5).

**Table 5:** Comparison of regression constants among various population

Study population	Arch	Sex	*r*	*r* ^*2*^	Regression coefficient		Standard error of mean (mm)
					a	b	
Thai [21]	Maxilla	M + F	0.60	0.36	11.87	0.47	0.84
	Mandible	M + F	0.64	0.41	10.3	0.5	0.82
Black American [50]	Maxilla	M + F	0.62	0.38	11.93	0.44	
	Mandible	M + F	0.70	0.49	9.93	0.52	
Negros [51]	Maxilla	M + F	0.65	0.42	10.18	0.52	0.87
	Mandible	M + F	0.70	0.49	8.30	0.64	0.94
Hong Kong Chinese [52]	Maxilla	M	0.79	0.62	7.79	0.66	0.68
		F	0.65	0.42	8.30	0.61	0.81
	Mandible	M	0.77	0.60	8.82	0.58	0.61
		F	0.69	0.47	6.66	0.64	0.82
Senegalese [43]	Maxilla	M + F	0.68	0.46	9.87	0.53	0.71
	Mandible	M + F	0.73	0.54	5.67	0.70	0.81
Tanaka Johnston [13]	Maxilla	M + F	0.63	0.40	10.41	0.51	0.86
	Mandible	M + F	0.65	0.42	9.18	0.54	0.85
Pakistani [20]	Maxilla	M + F	0.59	0.35	10.52	0.48	0.82
	Mandible	M + F	0.65	0.42	8.56	0.54	0.79
Kenyan [48]	Maxilla	M + F	0.75	0.56	6.55	0.68	0.91
	Mandible	M + F	0.61	0.37	11.33	0.48	0.95
Present Study	Maxillary	M	0.68	0.46	8.91	0.56	0.91
		F	0.77	0.59	3.65	0.78	0.91
	Mandibular	M	0.77	0.60	4.60	0.73	0.88
		F	0.71	0.50	5.58	0.67	0.93

*r* Correlation coefficient, *r* ^*2*^ Coefficient of determination, *M* Male, *F* Female

The magnitude of a standard error of the estimate (mean) is inversely proportional to the number of observations (that is the larger the sample size, the smaller the SEE). Therefore, it may not be reasonable to use this parameter in comparison of the present study to other studies due to difference in sample size. The regression line provided a mean predicted size of canine-premolars for a given size of the combined mesiodistal dimension of the permanent mandibular incisors.

Pearson product moment correlation is independent of both scales of measurement and sample size, and was therefore used for comparison of the predicted equations. A correlation coefficient that is equal to or greater than 0.6 is usually considered to be clinically significant.

When the measured value of the mesiodistal dimensions of canine and premolars are compared with the prediction equation given by Jaiswal et al. for Nepalese population, significant differences were found (Table 6).

**Table 6:** Comparison of Jaiswal prediction and measured sum of canine and premolars

	Mean	Std. deviation	Std. error mean	95 % Confidence interval of the difference			
				Lower	Upper	t	Sig. (2-tailed)
Jaiswal prediction -maxillary	0.44	1.02	0.10	0.24	0.65	4.36	0.00
Jaiswal prediction- mandibular	0.55	1.05	0.10	0.34	0.76	5.24	0.00

## Conclusions

1. The prediction equations of Tanaka-Johnston and the charts of Moyers (50 % or 75 % level of probability) did not accurately predict the mesiodistal dimension of unerupted canines and premolars in a sample of Nepalese Brahmins/Chhetris.
2. The regression equations proposed in this study are a good prediction method to determine the width of the maxillary and mandibular permanent canine and premolars in Nepalese Brahmins/Chhetris.
3. Further studies based on larger sample sizes are required to confirm the applicability of the proposed regression equations.

## Abbreviations

∑ CP1P2, sum of mesiodistal dimension of canine and two premolar; F, female; ICC, intraclass correlation coefficient; LI, sum of mesiodistal dimension of lower incisors; M, male; r, correlation coefficient; r^2^, coefficient of determination; SD, standard deviation; SEE, standard error of estimate; SEM, standard error of mean

## References

[1] Lee-Chan S, Jacobson BN, Chwa KH, Jacobson RS (1998). Mixed dentition analysis for Asian-Americans. Am J Orthod Dentofacial Orthop.

[2] Bishara SE, Jakobsen JR (1998). Comparison of two nonradiographic methods of predicting permanent tooth size in the mixed dentition. Am J Orthod Dentofacial Orthop.

[3] Hukaba G (1964). Arch size analysis and tooth size prediction. Dent Clin North Am..

[4] Nance HN (1947). Limitation of orthodontic treatment. I Diagnosis and treatment. Am J Orthod Oral Surg.

[5] Staley RN, Hoag JF (1978). Prediction of the mesiodistal tooth width of maxillary permanent canines and premolars. Am J Orthod..

[6] Hixon EH, Oldfather RE (1958). Estimation of the sizes of unerupted cuspid and bicuspid teeth. Angle Orthod..

[7] Staley RN, Kerber PE (1980). A revision of the Hixon and Oldfather mixed-dentition prediction method. Am J Orthod.

[8] Staley RN, O’Gorman TW, Hoag JF, Shelly TH (1984). Prediction of the widths of unerupted canines and premolars. J Am Dent Assoc.

[9] Staley RN, Shelly TH, Martin JF (1979). Prediction of lower canine and premolar widths in the mixed dentition. Am J Orthod.

[10] Irwin RD, Herold JS, Richardson A (1995). Mixed dentition analysis: a review of methods and their accuracy. Int J Paediatr Dent.

[11] Proffit WR, Fields HW, Sarver DM (2007). Contemporary Orthodontics.

[12] Moyers (1988). Handbook of Orthodontics.

[13] Tanaka MM, Johnston LE (1974). The prediction of the size of unerupted canines and premolars in a contemporary orthodontic population. J Am Dent Assoc.

[14] Dasgupta B, Zahir S (2012). Comparison of two non-radiographic techniques of mixed dentition space analysis and evaluation of their reliability for Bengali population. Contemp Clin Dent.

[15] Bugaighis I, Karanth D, Elmouadeb H (2013). Mixed dentition analysis in Libyan schoolchildren. J Orthod Sci.

[16] Paredes V, Tarazona B, Zamora N, Cibrian R, Gandia JL (2015). New regression equations for predicting human teeth sizes. Head Face Med.

[17] Sherpa J, Sah G, Rong Z, Wu L (2015). Applicability of the Tanaka–Johnston and Moyers mixed dentition analyses in Northeast Han Chinese. J Orthod.

[18] Manjula M, Rani S, David S, Reddy E, Sreelakshmi N, Rajesh A (2013). Applicability of tooth size predictions in the mixed dentition space analysis in Nalgonda population. J Dr NTR Univ Health Sci.

[19] Ramesh N, Reddy MSR, Palukunnu B, Shetty B, Puthalath U (2014). Mixed Dentition Space Analysis in Kodava Population: A Comparison of Two Methods. J Clin Diagn Res.

[20] Bherwani AK, Fida M (2011). Development of a prediction equation for the mixed dentition in a Pakistani sample. Am J Orthod Dentofacial Orthop.

[21] Jaroontham J, Godfrey K (2000). Mixed dentition space analysis in a Thai population. Eur J Orthod.

[22] Al-Bitar ZB, Al-Omari IK, Sonbol HN, Al-Ahmad HT, Hamdan AM (2008). Mixed dentition analysis in a Jordanian population. Angle Orthod.

[23] al-Khadra BH (1993). Prediction of the size of unerupted canines and premolars in a Saudi Arab population. Am J Orthod Dentofacial Orthop.

[24] Alzubir AA, Abass S, Ali MA (2016). Mixed dentition space analysis in a Sudanese population. J Orthod.

[25] Srivastava B, Bhatia H, Singh R, Singh A, Aggarwal A, Gupta N (2013). Validation of Tanaka and Johnston’s analysis in western UP Indian population. J Indian Soc Pedod Prev Dent.

[26] Jaiswal AK, Paudel KR, Shrestha SL, Jaiswal S (2009). Prediction of space available for unerupted permanent canine and premolars in a Nepalese population. J Orthod.

[27] Nepal population report 2011 (2011). Ministry of Health and population, Government of Nepal.

[28] Altman DG (1980). Statistics and ethics in medical research: III How large a sample?. Br Med J.

[29] Richardson ER, Malhotra SK (1975). Mesiodistal crown dimension of the permanent dentition of American Negroes. Am J Orthod.

[30] Bishara SE, Jakobsen JR, Abdallah EM, Fernandez Garcia A (1989). Comparisons of mesiodistal and buccolingual crown dimensions of the permanent teeth in three populations from Egypt, Mexico, and the United States. Am J Orthod Dentofacial Orthop.

[31] Shrestha R (2005). Measurement of mesio-distal tooth diameter of Nepalese permanent dentition. J Nep Dent Assoc.

[32] Hunter WS, Priest WR (1960). Errors and discrepancies in measurement of tooth size. J Dent Res..

[33] Johnston LE (2002). Regression: Is your guess as good as mine?. Semin Orthod.

[34] Howe RP, McNamara JA, O’Connor KA (1983). An examination of dental crowding and its relationship to tooth size and arch dimension. Am J Orthod.

[35] Puri N, Pradhan KL, Chandna A, Sehgal V, Gupta R (2007). Biometric study of tooth size in normal, crowded, and spaced permanent dentitions. Am J Orthod Dentofacial Orthop.

[36] Garn SM, Lewis AB, Kerewsky RS (1966). The meaning of bilateral asymmetry in the permanent dentition. Angle Orthod.

[37] Bishara SE, Garcia AF, Jakobsen JR, Fahl JA (1986). Mesiodistal crown dimensions in Mexico and the United States. Angle Orthod.

[38] Seipel C (1946). Variation in tooth position: a metric study of variation and adaptation in the deciduous and permanent dentitions. Swed Dent J.

[39] Doris JM, Bernard BW, Kuftinec MM, Stom D (1981). A biometric study of tooth size and dental crowding. Am J Orthod.

[40] Arya BS, Savara BS, Thomas D, Clarkson Q (1974). Relation of sex and occlusion to mesiodistal tooth size. Am J Orthod.

[41] Axelsson G, Kirveskari P (1983). Crown size of permanent teeth in Icelanders. Acta Odontologica.

[42] Yuen KK, So LL, Tang EL (1997). Mesiodistal crown diameters of the primary and permanent teeth in southern Chinese--a longitudinal study. Eur J Orthod.

[43] Diagne F, Diop-Ba K, Ngom PI, Mbow K (2003). Mixed dentition analysis in a Senegalese population: elaboration of prediction tables. Am J Orthod Dentofacial Orthop.

[44] Ballard ML, Wylie WL (1947). Mixed dentition case analysis, estimating size of unerupted permanent teeth. Am J Orthod.

[45] Arslan SG, Dildes N, Kama JD, Genc C (2009). Mixed-dentition analysis in a Turkish population. World J Orthod.

[46] Burhan AS, Nawaya FR (2014). Prediction of unerupted canines and premolars in a Syrian sample. Prog in Orthod..

[47] Buwembo W, Kutesa A, Muwazi L, Rwenyonyi CM (2012). Prediction of width of un-erupted incisors, canines and premolars in a Ugandan population: a cross sectional study. BMC Oral Health.

[48] Ngesa JL (2004). Applicability of tooth size predictions in the mixed dentition analysis in a Kenyan sample: University of the Western cape.

[49] Melgaco CA, Araujo MT, Ruellas AC (2006). Applicability of three tooth size prediction methods for white Brazilians. Angle Orthod.

[50] Frankel HH, Benz EM (1986). Mixed dentition analysis for black Americans. Pediatr Dent.

[51] Ferguson FS, Macko DJ, Sonnenberg EM, Shakun ML (1978). The use of regression constants in estimating tooth size in a Negro population. Am J Orthod.

[52] Yuen KK, Tang EL, So LL (1998). Mixed dentition analysis for Hong Kong Chinese. Angle Orthod.

